# Olympic Win: Lower Estimated Cancer Risk with Air Pollution Controls during the 2008 Beijing Games

**DOI:** 10.1289/ehp.119-a259b

**Published:** 2011-06

**Authors:** Julia R. Barrett

**Affiliations:** **Julia R. Barrett** a Madison, WI–based science writer and editor, has written for *EHP* since 1996. She is a member of the National Association of Science Writers and the Board of Editors in the Life Sciences

Polycyclic aromatic hydrocarbons (PAHs) sorbed to fine atmospheric particulate matter (PM_2.5_) increase inhalation cancer risk in exposed populations. In China each year, an estimated 6.5 people per million develop lung cancer due to PAH inhalation. But stringent air pollution control measures instituted during the 2008 Olympic Games in Beijing, if sustained over a lifetime, could reduce residents’ PAH-related inhalation cancer risk by nearly half **[*EHP*** **119(6):815–820; Jia et al.]**.

Coal combustion and motor vehicle emissions create severe air pollution in Beijing and other major Chinese cities. To improve air quality for the Olympic Games, factories in and around Beijing were moved or closed, vehicular traffic was restricted, and truck traffic was reduced during the period 20 July–20 September 2008. During the games themselves (8–24 August), even stricter controls were imposed.

PM_2.5_ samples were collected at a single representative location in Beijing, and associated concentrations of 17 PAHs were measured and compared for four periods: 1) 28 July–20 September versus 2) 21 September–7 October, and 3) 8–24 August versus 4) 28 July–7 August/25 August–7 October. To enable direct comparison of chemical risks, a benzo[*a*]pyrene equivalent (BaP_eq_) concentration was estimated for each PAH by multiplying its concentration by its relative potency factor (RPF). Cancer risk was calculated using the BaP_eq_ values paired with previously established unit risk measures for cancer based on a lifetime (70 years) of BaP exposure at 1 μg/m^3^ air.

Individual BaP_eq_ concentrations were 22–78% lower in period 1 compared with period 2 (i.e., when any pollution controls were in place) and 32–72% lower in period 3 compared with period 4 (i.e., when the strictest controls were in place). Lifetime excess inhalation cancer cases estimated during the period when pollution was controlled ranged from 6.5 to 518 individuals per million compared with a range of 12.2–964 per million after pollution controls ceased—a 46% reduction. PAHs from the U.S. Environmental Protection Agency’s list of priority pollutants made up three of the four largest contributors to total carcinogenicity; however, high-molecular-weight PAHs—a highly carcinogenic group of chemicals that, as a class, haven’t been extensively studied—contributed a considerable 23% of the cancer risk. These top four PAHs are primarily associated with PM_2.5_, so reducing PM_2.5_ emissions would also reduce levels of these and other PAHs.

Limitations of the study include potential inaccuracies due to its point-estimate approach, which assumes additive cancer risk, and the RPF values, which were based on toxicologic studies with their own uncertainties. However, the study strongly supports the effectiveness of air pollution source control measures and also demonstrated the need to include high-molecular-weight PAHs in future studies.

## Figures and Tables

**Figure:**
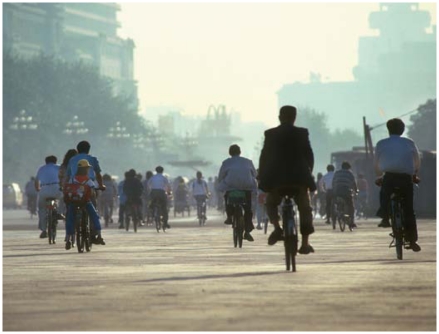
Early morning rush hour, Tiananmen Square, Beijing

